# Comparison of physical effect between two training methods for individuals with substance use disorder

**DOI:** 10.1186/s13102-021-00234-y

**Published:** 2021-01-21

**Authors:** Yang Yan-guang, Chen Jing-yi, Pang Xiao-Wu, Shen Meng-lu, Yang Su-yong, Xu Ding, Xiao Ke, Wang Tian-yuan, Wang Jia-bin, Zhu Dong

**Affiliations:** 1 Wushu College, Shanghai University of Sport, 200438 Shanghai, China; 2 School of Sport Psychology, Shanghai University of Sport, 200438 Shanghai, China; 3 Shanghai Drug Administration, 200080 Shanghai, China; 4 Shanghai Gaojing Drug Compulsory Detoxification and Rehabilitation Center, 200439 Shanghai, China; 5 School of International Education, Shanghai University of Sport, 200438 650 Qing Yuan Huan road, Yang Pu District, Shanghai, China

**Keywords:** High‐intensity interval training, Moderate‐intensity continuous training, Substance use disorder, Physical fitness, Amphetamine‐type stimulants, Tai chi

## Abstract

**Background:**

HIIT has recently been widely used for health promotion in healthy people and patients with chronic diseases. Exercise can help SUD reduce drug cravings, enhance mental health and return to normal life. However, whether HIIT can bring better physical rehabilitation benefits to individuals with SUD than MICT is unclear. The study aimed to compare the effects of HIIT versus MICT on the physical fitness of individuals with SUD.

**Methods:**

One hundred twenty individuals with amphetamine-type stimulant dependence voluntarily participated in this study. They were randomly assigned to the HIIT group and MICT group. Both groups received training three times a week. The intervention lasted from January 2019 to December 2019. Physical fitness was assessed at the baseline, 3 months, 6 months, 9 months and 12 months, including blood pressure (BP), vital capacity(VC), hand grip, push-up, sit-and-reach, one-leg standing with eyes closed and choice reaction time. The craving level was assessed using the Visual Analog Scale at baseline, 6 months and 12 months to see any change along with the improvement in physical fitness. Two-way repeated measures ANOVA was applied to analyse the differences in change by group (HIIT and MICT) and time (baseline, 3 months, 6 months, 9 months and 12 months).

**Results:**

The within-group factor displayed significant changes in the HIIT and MICT groups in terms of systolic BP (F _(4,336)_ = 12.799, *P* < 0.001,η2 = 0.204), diastolic BP (F_(4,336)_ = 9.495, *P* < 0.001, η2 = 0.16), VC (F_(4,336)_ = 18.121, *P* < 0.001, η2 = 0.177), hand grip (F_(4,336)_ = 34.815, *P* < 0.001, η2 = 0.293), sit-and-reach (F_(4,336)_ = 13.871, *P* < 0.001, η2 = 0.142), push-up (F_(4,336)_ = 28.805, *P* < 0.001, η2 = 0.255), one-leg standing with eyes closed (F_(4,336)_ = 14.495, *P* < 0.001, η2 = 0.156) and choice reaction time (F_(4,336)_ = 20.603, *P* < 0.001, η2 = 0.197). The craving level decreased after 12 months of intervention in both groups (F_(2,168)_ = 11.25, *P* < 0.001, η2 = 0.118), but no significant differences in physical fitness and craving level were found in between groups and the interactions of group × time.

**Conclusions:**

After 12 months of intervention, physical fitness improved while craving level decreased in the two groups. These findings suggest that both HIIT and MICT have positive effects on individuals with SUD in terms of physical fitness.

**Trial registration:**

ChiCTR1900022158 Chinese Clinical Trial Registry: Registered 27th March, 2019.

## Background

Substance use disorder (SUD) is a highly destructive, chronic and relapsing disease that brings adverse consequences to society and the individuals with SUD, requiring more effective treatment methods [1]. The social harm caused by the illicit drug problem is huge. At the same time, helping individuals rehabilitate from drug abuse and return to normal life has become an important field of rehabilitation. As a result of long-term drug abuse, individuals with SUD suffer from serious physical and psychological damage, leading to the occurrence of various chronic diseases and complications [2]. Individuals with SUD have less muscle protein and mass than healthy individuals, resulting in hypertension reflexes, dyskinesia and gait instability [3]. In addition, SUD may lead to a decline in physical fitness, tachycardia, high blood pressure (BP) and chronic cardiovascular diseases [4]. Physical and mental injuries affect the quality of life of patients with SUD but also have a negative impact on detoxification. Effective physical health education and exercise guide are the best carriers to promote the integration of an individual’s body and mind, to realise ‘strengthening the body and civilised the spirit’, to improve the recovery rate of drug withdrawal and to reduce the relapse rate [5]. A study showed that exercise may serve to complement other therapy and medication approaches for methamphetamine users, particularly if users are able to more readily utilise relapse prevention skills and incorporate positive behavioural changes consistent with treatment goals [6]. Currently, exercise is considered a potential new treatment for SUD, and exercise intervention is also regarded as an independent and important supplemental means for SUD [7]. Exercise has become one of the promising intervention methods for SUD because it is economical and produces long-term effects.

A growing body of research has demonstrated that aerobic exercise can be an effective and persistent treatment for those with SUD, which can effectively increase the abstinence rate, ease withdrawal symptoms and reduce anxiety and depression [8]. Moderate-intensity aerobic exercise has shown good rehabilitation benefits in assisting detoxification, relieving withdrawal syndrome and inhibiting relapse impulse and relapse behaviour, and it is considered relatively safe and green [9]. Furthermore, a large number of theoretical and empirical studies have found that short-term, medium-intensity aerobic exercise, traditional Chinese sports and resistance exercise can significantly improve the cognition, psychological behaviour and physical function of drug dependents, improve their quality of life and reduce drug cravings [10]. In terms of psychological rehabilitation, many studies have shown that exercise can produce positive emotions for drug users, including regulating emotions, experiencing pleasure and reducing depression symptoms and insomnia [11].

Although the mechanism of exercise detoxification is not clear, it does bring benefits to individuals with SUD. Studies have shown that exercise can improve executive function and brain activity [12], which may be an important reason to curb addiction in individuals with SUD. To further enhance the training effect, the most suitable exercise mode should be explored. To date, many articles on drug rehabilitation have described the benefits of moderate-intensity continuous training (MICT). However, future research in the field of exercise intervention should focus on using anaerobic exercise as an intervention to explore whether there is a dose-effect relationship to inhibit relapse [13]. Many studies compared the effect of high-intensity interval training (HIIT) and MICT, and research indicates that HIIT has better effects on cognitive ability and cardiopulmonary function than MICT [14]. The results of sports studies showed that MICT and HIIT are both beneficial but have different effects on the improvement of body function [15]. Moreover, meeting the minimum 2008 Physical Activity Guidelines for Americans by either moderate- or vigorous-intensity activities is associated with nearly the maximum longevity benefit [16].

Although people pay attention to MICT exercise rehabilitation, the emergence of HIIT has attracted much concern for its time saving and favourable effect. The improvement in mental and cognitive health due to HIIT, compared with traditional high-intensity sports, as an effective way to improve physical health, has also attracted great interest in recent years [17].

Increasing evidence showed that despite a reduction in exercise duration and volume, HIIT results in similar or greater physical adaptations relative to MICT, including improvements in body composition, cardiovascular function and metabolic health [18]. Compared with the traditional moderate-intensity continuous aerobic training, HIIT has better exercise enjoyment and provides the same effects in terms of aerobic fitness, quality of life, efficiency, safety, tolerance and short-term exercise adherence [19].

HIIT not only brings more effective training to healthy people but is also an important means for the rehabilitation of many chronic diseases. In recent years, HIIT has become a form of alternative or complementary aerobic endurance training. In long-term and short-term studies, it has the same value as, if not better than, MICT in terms of fitness, cardiovascular function, quality of life, exercise efficiency, safety, tolerance and exercise adherence within a short time period [20]. A meta-analysis of 10 studies on patients with coronary artery disease showed that HIIT is more effective in improving patients’ mean peak oxygen uptake (VO_2_ peak) than MICT [21]. Studies have confirmed that HIIT can effectively prevent and treat type 2 diabetes and cardiovascular disease, and it has great potential in the field of public health [22]. A previous study also found that HIIT can improve the maximum oxygen uptake (VO_2_ max) of patients with SUD, indicating that not only will they have a strongly decreased mortality rate but also a considerable reduced risk of developing cardiovascular disease [23]. Given its time-saving and remarkable effect, HIIT has gradually become an alternative to the traditional training mode, and it has achieved good results. An acute bout of HIIT can also be more enjoyable than an acute bout of MICT [24]. Notably, sedentary young adults report greater enjoyment from a single bout of HIIT and endorse it as an exercise regime they would chose to continue on their own [24]. A six-week long-term HIIT and MICT trial for sedentary people also showed that the subjects’ enjoyment of HIIT increased while the pleasure of MICT decreased slightly [19]. These findings showed that HIIT has better exercise enjoyment than MICT.

To date, only few articles in the literature discussed the effect of HIIT on SUD. One showed that HIIT is feasible for patients with SUD in treatment, whereas another reported that HIIT can improve depression and reduce the risk of cardiovascular disease [23]. However, the theoretical basis for adopting HIIT has not been fully confirmed in scientific reports. The effects of MICT on the physical functioning of individuals with SUD have been demonstrated, but whether different intensities or forms of exercise produce the same or superior results remains unclear. Considering the benefits and theoretical results of HIIT in the study of many chronic diseases, it is feasible to apply it in SUD. Therefore, this study proposes the following question: which exercise is better for SUD: HIIT or MICT? The purpose of this study was to compare the physical fitness of HIIT or MICT intervention amongst individuals with SUD. This study hypothesised that HIIT has better physical fitness recovery effect than MICT for SUD.

## Methods

### Design

This single-blind (assessors-blind), two-group randomised controlled trial was conducted from January 2019 to December 2019. The study protocol was approved by the Ethical Committees of the Shanghai University of Sport and the Shanghai Narcotics Control Commission. Written consent was obtained from each participant.

### Participants

The participants were all male amphetamine-type stimulant (ATS)-dependent individuals. At the time of recruitment, a total of 1200 individuals with SUD were receiving treatment in a Shanghai Compulsory Rehabilitation Centre (SCRC). A total of 120 ATS-dependent subjects voluntarily participated in this study. The inclusion criteria were (1) age 18–40; (2) subjects who met the diagnosis of methamphetamine (MA) dependence according to the Diagnostic and Statistical Manual of Mental Disorders Criteria (DSM-IV); (3) the treatment duration in SCRC should be more than 1 year; (4) no serious medical or mental illness; and (5) educational attainment of primary school or above. Exclusion criteria were as follows: (1) currently diagnosed diseases of the cardiovascular system, respiratory system and nervous system; (2) anti-social personality disorder and borderline personality disorder; and (3) subjects who were unwilling to accept the assigned intervention conditions.

### Procedure

Participants recruited from SCRC were randomly assigned by computer-generated randomisation to either the HIIT (n = 60) or MICT groups (n = 60). Participants in both groups received a 1 h intervention 3 times per week for 12 months. The HIIT exercise sessions were administered in the form of circuit training with a work-to-recovery ratio of 1:0.5, whilst MICT was carried out without recovery sessions for the TC group. They all performed the intervention under the supervision of professional instructors on a basketball field under fair weather or in an indoor fitness gym. The interventions were conducted every Monday, Wednesday and Friday. The outcomes of physiological and physical fitness including BP, vital capacity (VC), push-up, sit-and-reach, one-leg standing with eyes closed, choice reaction time (CRT) and hand grip were assessed at the baseline, 3 months, 6 months, 9 months and 12 months. The ATS craving level was assessed at the baseline, 6 months and 12 months. The experienced researchers conducted the assessment and were blinded to the two groups. All subjects voluntarily participated in this experiment, and they signed the informed consent prior to the study. The study was performed in accordance with the Declaration of Helsinki II. Participants received no compensation during the intervention, but extra nutritious food was provided on the training day.

### Intervention

### HIIT group

The training contents in the HIIT group included non-confrontational basketball training, resistance training (weight training and strength machines), rope skipping and running. The duration of each training session was 60 min, including 10 min of warm-up, 40 min of HIIT session and 10 min of cool down. In each training session, subjects were asked to complete the HIIT session under the training and recovery ratio of 1:0.5. The exercise intensity was monitored by using a heart rate (HR) monitor (Polar TeamPro), and the HR was maintained at 80–85 % of the subjects’ maximum HR (80–85 % HRmax) under the supervision of professional instructors. The average HR was maintained at 150–160 beats per minutes during the HIIT training session and 130–140 beats during the recovery session. The training patterns were short interval training organised by the experienced instructors from Shanghai University of Sports. An exercise training expertise team instructed and supervised the training plan of the HIIT group.

### MICT group

The MICT group was trained with Tai Chi, mind-body exercise and recreational activity. The average HR during exercise was maintained at 105–125 beats per minute according to the HR monitor (Polar TeamPro) under the supervision of an instructor which obtained 55–65 % HRmax. The duration of the MICT intervention was similar to that of the HIIT intervention, and the training intervention of the two groups was conducted at the same time. Tai Chi for the MICT group was a kind of modified Tai Chi. The recreational activity adopted the ninth edition of ‘Guang Bo Ti Cao’ designed by the China General Administration of Sports, whilst the mind-body exercise adopted a kind of modified Qi Gong, Tai Chi and Yoga with moderate intensity. Each session in the MICT group included 10 min of warm-up, 10 min of recreational activities (Guang Bo Ti Cao), 10 min of mind-body exercise, 20 min of Tai Chi and 10 min of cool down. The duration of Tai Chi in a training session was increased to more than 20 min in the second half of the year, along with the reduction of the training duration of mind-body exercise and recreational activity. One experienced instructor from the Shanghai University of Sport instructed the MICT group.

### Outcome measures

#### Primary outcomes

The physical fitness tests were administered by experienced investigators. Measurements were performed in the morning at the same time. BP was measured under standardised conditions prior to other tests: participants were asked to rest for 5 min and had not taken any caffeine or tobacco products within 30 min. The HR of individuals with SUD was monitored with Polar TeamPro.

The fitness test followed the protocol of the national physical fitness test. Push-up, sit-and-reach, one-leg standing with eyes closed, VC, height and weight, CRT and hand grip were assessed using the model of BW-FC-9201L (Fitness Assessment System) to determine the physical fitness outcomes. All tests were repeated twice, and the best performance was recorded. VC measurement requires that the subject holds the test instrument and breathes out all the air with maximum force after the maximum inhalation with millilitres (ml). Muscle strength tests include hand grip and push-up. During the hand grip test, subjects were instructed to grasp the grip strength device for a few seconds with their maximum strength indicated by kilograms (kg). The push-up test was performed on the push-up testers, with subjects’ forefoot or toes on floor, hips and back straight and the push-up tester placed near the subject’s chest. The repetition numbers were counted automatically by the push-up tester whenever the torso move down and up front of the tester sensors. The test index of flexibility was sit and reach. Subjects were asked to sit in front of the device with slippers and knees straight and stretch their hands and body forward as far as possible. Maximum distance was measured in centimetres (cm) where subjects’ fingertips could reach. In the choice reaction time test, the subjects used the middle and index fingers of the dominant hand to quickly press the random signal button. The results were evaluated in terms of time in milliseconds (ms). Balance function was measured by the duration of standing on one leg with eyes closed in seconds (s). During the test, the subjects stood on the test mat with the dominant leg, eyes closed; the time of standing on one leg until any part of subject’s body touched the test mat was recorded.

#### Secondary outcome

The craving level was assessed using the visual analogue scale (VAS), which is a response scale that measures subjective attitudes that cannot be observed directly. Subjects were asked to indicate, on a scale from 0 (no craving for ATS drugs) to 10 (strong craving for ATS drugs), the extent of their craving for ATS drugs. A VAS is considered an appropriate means of assessing cue-induced ATS craving [25].

### Statistical analyses

Statistical analyses were performed using SPSS 22.0 (Chicago, IL, USA). Pearson chi-squared test was applied for categorical variables of demography, and independent sample t-test was applied for continuous variables at the baseline comparison. Pearson chi-squared test and independent sample t-test were used to compare the differences in demographic characteristics at baseline.

The normal distribution of variables was tested with the Kolmogorov–Smirnov test, and two-way repeated measures ANOVA was applied to test whether the treatments differ after 12 months. Time (baseline, 3 months, 6 months, 9 months and 12 months) was the within-group factor, and groups (HIIT and MICT) were the between-group factors for the physical fitness comparison. Time (baseline, 6 months and 12 months) was the within-group factor, and groups (HIIT and MICT) were the between-group factors for the craving level of VAS comparison. A post-hoc test with Bonferroni correction was used to examine differences in the baseline and 3 months, 6 months, 9 months and 12 months when ANOVA showed a significant interaction. Effect size was used with the partial eta square as η^2^. All data except the post-hoc test were presented as the mean ± standard deviation, and P < 0.05 was considered statistically significant. The data of the post-hoc test were presented as the mean difference ± standard error, and statistical significance was set at *P* < 0.05.

Data that failed to pass the Kolmogorov–Smirno test were subjected to the non-parametric test with Mann–Whitney U test.

## Results

**Fig. 1:**
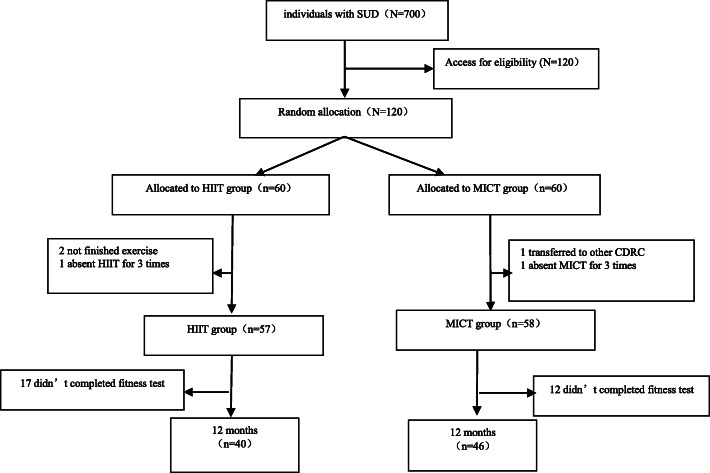
Flow diagram of intervention progress through the phases of the two groups. (SUD: Substance Use Disorder; HIIT: High-intensity Interval Training; MICT: Moderate-intensity Continuous Training; CDRC: Compulsory Detoxification and Rehabilitation Centre)

A total of 120 male individuals with SUD participated in the study. Of these individuals, 86 completed the intervention, and 34 subjects dropped out because of different reasons (Fig. 1).

The subjects in the HIIT group had the following demographics: age, 33.65 ± 4.27 years; height, 172.69 ± 5.65 cm; weight, 76.05 ± 10.95 kg; and years of illicit drug use, 8.90 ± 5.22 years. Meanwhile, subjects in the MICT group had the following demographics: age, 32.20 ± 5.07 years; height, 174.36 ± 5.70 cm; weight, 76.33 ± 9.54 kg; and years of illicit drug use, 7.14 ± 4.53 years. At baseline, the independent T-test result of the physical fitness and craving level of VAS between the two groups showed no significant difference (*P* > 0.05; Table 1).

**Table 1:** Baseline demographic characteristics of participants

	HIIT group		MICT group		T-value
	(*n* = 60)		(*n* = 60)		
	Means	SD	Means	SD	
Height (cm)	172.69	5.65	174.36	5.71	−1.587
Weight (kg)	76.05	10.95	76.34	9.54	− 0.151
Age (years)	33.65	4.27	32.2	5.07	1.806
Year of illicit drug use (years)	8.9	5.22	7.14	4.53	0.916
Systolic BP (mmHg)	127.9	16.22	128.22	19.41	− 0.098
Diastolic BP (mmHg)	74.9	12.36	73.41	14.64	0.594
Vital capacity (ml)	3983.74	793.97	4116.93	629.42	−1.006
Hand grip (kg)	47.24	7.55	48.08	7.79	− 0.596
Push up (Rep)	26.26	18.703	21.66	13.23	1.537
Sit-and-reach (cm)	15.11	6.4	15.57	6.81	− 0.380
One-leg stand with eyes closed (s)	23.71	17.2	22.82	17.67	0.278
Choice reaction time (ms)	0.5409	0.0994	0.5331	0.0728	0.485
Craving level of VAS	2.16	1.57	2.54	1.82	−1.028

Data were analysed with independent T test; *, p < 0.05; **, P < 0.01

*HIIT* High-intensity Interval Training; *MICT* Moderate-intensity Continuous Training; *BP* Blood Pressure; *VAS* Visual Analogue Scale

### Physiological outcomes

The results analysed using repeated measures ANOVA indicated significant differences in the within-group factor in terms of systolic blood pressure (SBP) (F _(4,336)_ = 12.799, *P* < 0.001,η^2^ = 0.204), diastolic blood pressure (DBP) (F_(4,336)_ = 9.495, *P* < 0.001, η^2^ = 0.16) and VC (F_(4,336)_ = 18.121, *P* < 0.001, η^2^ = 0.177) after 12 months of exercise intervention. There was no significant difference between groups and interactions of group × time in SBP, DBP and VC.

### Physical fitness

Significant differences were found in the within-group factor of hand-grip power (F_(4,336)_ = 34.815, *P* < 0.001, η^2^ = 0.293), sit-and-reach (F _(4,336)_ = 13.871, *P* < 0.001, η^2^ = 0.142), push-up (F _(4,336)_ = 28.805, *P* < 0.001, η^2^ = 0.255), one-leg standing with eyes closed (F_(4,336)_ = 15.495, *P* < 0.001, η^2^ = 0.156) and CRT (F _(4,336)_ = 20.603, *P* < 0.001, η^2^ = 0.197) tests.

**Fig. 2:**
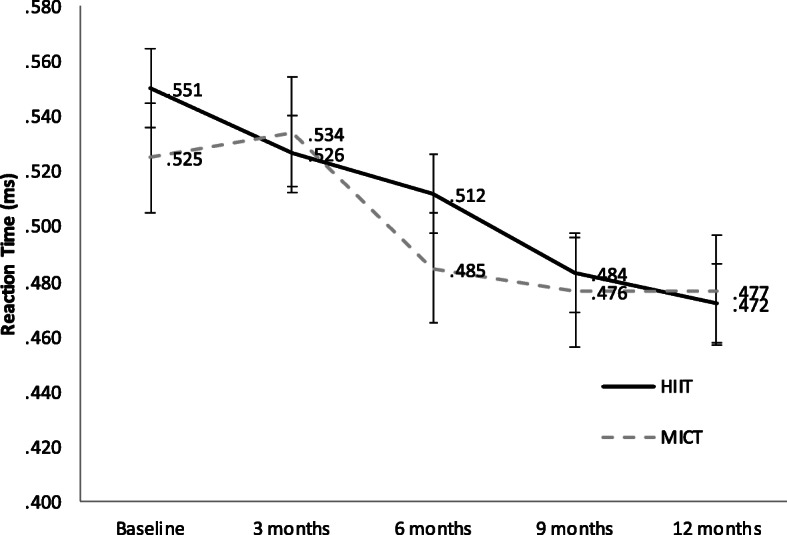
Comparison of choice reaction time between the HIIT and MICT groups. The solid line represents the HIIT group, and the broken line represents the MICT group. The grey line on the bottom indicates the exercise intervention period. Standard deviation is displayed as bars. (HIIT: High-intensity Interval Training; MICT: Moderate-intensity Continuous Training)

**Fig. 3:**
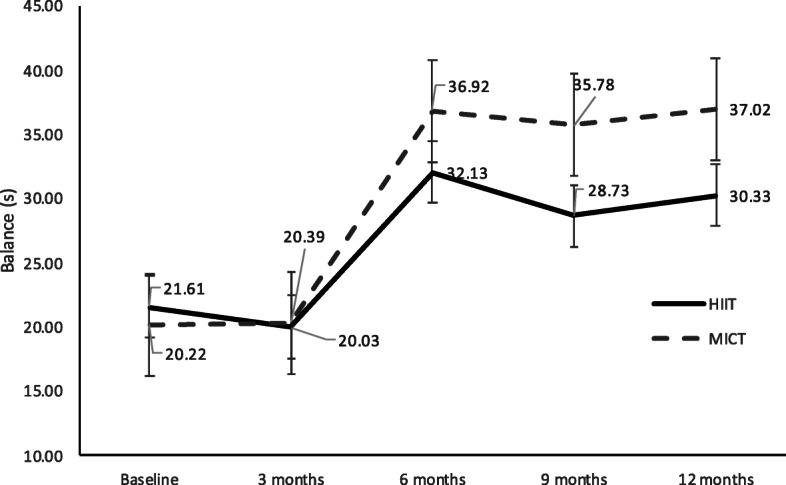
Balance comparison of the HIIT and MICT groups by one-leg standing with eyes closed. The solid line represents the HIIT group, and the broken line represents the MICT group. The grey line on the bottom indicates the exercise intervention period. Standard deviation is displayed as bars. (HIIT: High-intensity Interval Training; MICT: Moderate-intensity Continuous Training)

**Fig. 4:**
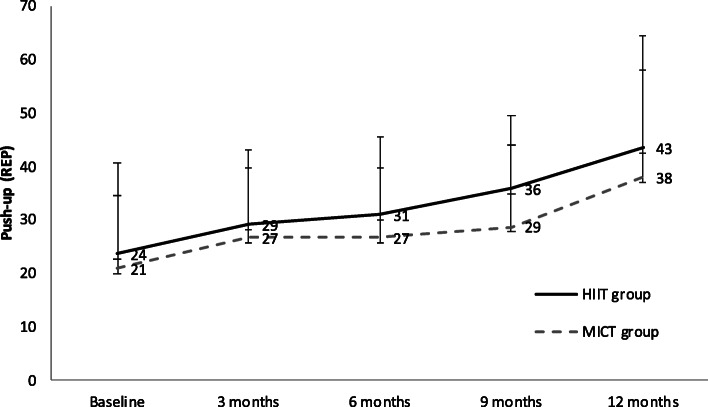
Comparison of push-ups between the HIIT and MICT groups. The solid line represents the HIIT group, and the broken line represents the MICT group. The grey line on the bottom indicates the exercise intervention period. Standard deviation is displayed as bars.(HIIT: High-intensity Interval Training; MICT: Moderate-intensity Continuous Training; REP: Repetitions)

Furthermore, the CRT decreased by 0.03 s in the experimental group and decreased by 0.04 s in the control group (Fig. 2). Although the balance in both groups improved from the beginning, the two groups showed an improvement in the first half of the year. However, the balance test by one-leg standing with eyes closed did not improve from the seventh month, indicating that the MICT group had a better balance score than the HIIT group (Fig. 3). Subjects in the HIIT and MICT groups showed a gradual improvement in the upper-limb strength due to push-ups, and the mean difference of subjects in the HIIT group was 4.4 more than subjects in the MICT group (Fig. 4).

No significant differences were found between groups and interactions of group × time in terms of hand-grip power, push-up, sit-and-reach, one-leg standing with eyes closed and CRT test (Table 2).

**Table 2:** Physical fitness comparison between the HIIT and MICT groups at baseline and 12 months

	HIIT (*n* = 40)		MICT (*n* = 46)		Within-group	Between-group	Time × Group
	Baseline	12 months	Baseline	12 months	*F* value	*F* value	*F* value
Systolic BP (mmHg)	129.40 ± 16.39	123.03 ± 15.34	124.32 ± 20.53	123.5 ± 16.28	12.799**	0.384	1.647
Diastolic BP (mmHg)	75.10 ± 11.75	70.90 ± 8.81	70.32 ± 11.94	71.55 ± 14.69	9.495**	0.796	1.665
Vital capacity (ml)	3891.53 ± 779.25	4475.83 ± 853.85	4192.93 ± 538.29	4647.39 ± 1032.35	18.121**	1.117	0.519
Grip strength (kg)	47.21 ± 7.24	51.75 ± 7.22	48.08 ± 7.72	52.23 ± 7.41	34.815**	0.315	0.058
Push up (Rep)	23.67 ± 17.08	43.40 ± 21.12	20.87 ± 13.65	37.03 ± 20.08	28.805**	3.017	0.587
Sit and reach (cm)	15.41 ± 5.92	15.88 ± 7.98	14.76 ± 6.55	13.97 ± 7.74	13.871**	1.654	0.277
One-leg stand with eyes closed (s)	21.61 ± 14.32	30.33 ± 24.09	21.22 ± 16.38	37.02 ± 27.75	15.495**	1.102	1.112
Choice reaction time (s)	0.55 ± 0.10	0.47 ± 0.06	0.52 ± 0.07	0.48 ± 0.06	20.603**	0.639	1.662

Data were analysed with repeated measures ANOVA, * displayed as F value < 0.05; ** displayed as F < 0.01

*HIIT* High-intensity Interval Training; *MICT* Moderate-intensity Continuous Training; *BP* Blood Pressure; *VAS* Visual Analogue Scale

### Level of craving

**Fig. 5:**
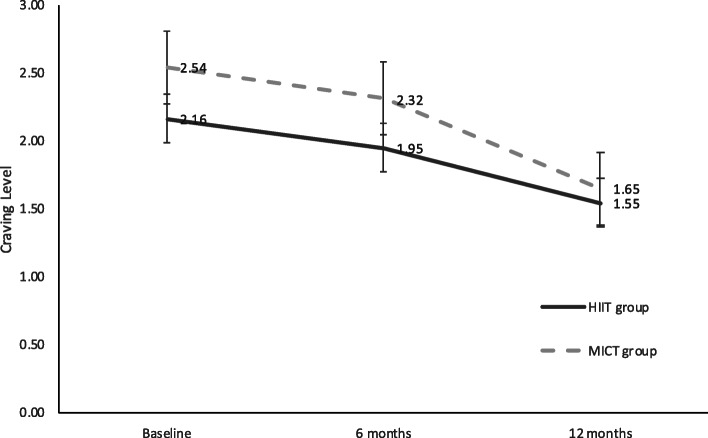
Craving level comparison of the HIIT and MICT groups. The solid line represents the HIIT group, and the broken line represents the MICT group. The grey line on the bottom indicates the exercise intervention period. Standard deviation is displayed as bars. (HIIT: High-intensity Interval Training; MICT: Moderate-intensity Continuous Training)

The craving level of VAS after 12 months of exercise intervention significantly decreased (F_(2,168)_ = 11.25, *P* < 0.001, η^2^ = 0.118) in both groups, but no significant differences with interactions of group × time were found in VAS (Fig. 5).

## Discussion

### Physical fitness improved in both groups

In this study, a randomised controlled trial of HIIT and MICT was conducted to explore whether HIIT is more conducive to the rehabilitation of physical functions for individuals with SUD than MICT. During 12 months of exercise intervention, the HIIT and MICT groups exhibited a significant improvement in VC, grip strength, push-up, sit-and-reach, one-leg standing with eyes closed and CRT. The BP and craving level in the two groups decreased significantly, but no significant differences were found between the HIIT and MICT groups.

### CRT declined

CRT is one of the most important indicators of physical fitness. It reflects the function of the human nervous system to some extent. The smaller the reaction time, the faster the human body responses, and the better the function of the nervous system is. Performance in a CRT task is applied as an indicator of cognitive capacity, including aspects of eye-hand reaction time, attentiveness and processing speed [26]. The results consistently showed that physical activity can improve CRT, which indicates that any form of exercise is helpful to improve the CRT.

### Balance improved

The one-leg standing with eyes closed test was performed to measure the balance control of the subjects. The increase in time spent standing on one foot with eyes closed in individuals with SUD indicated the improvement in their balance ability. A previous study showed an improvement in balance control after long-term practice of Tai Chi [27]. In this study, the MICT group was able to stand on one leg with eyes closed for 14 s longer than the baseline, while the HIIT group was only able to do so for 11 s longer than the baseline. These findings proved that both HIIT and MICT could improve balance ability. Although there was no significant difference between groups, the MICT group showed more potential to improve balance than the HIIT group because of the Tai Chi content.

### Blood pressure decreased

The subjects in the HIIT and MICT groups had the same effect on DBP and SBP; both types of training effectively reduced the BP for individuals with SUD. A reduction in BP can have a positive effect on the health of individuals. SBP is controlled by total peripheral resistance, which is determined by the diameter of small arteries and arterioles [27]. The decrease in BP in individuals with SUD may be due to increased flexibility in their blood vessels through training and reduced peripheral resistance. In this study, exercise intervention can significantly reduce BP, which may be due to Tai Chi and HIIT, which improved the cardiac function of subjects, as well as increase stroke output and blood circulation. The relaxation form of exercise may reduce the tension of the vascular centre and vascular smooth muscles and accelerate blood flow, leading to the reduction in BP. The practice of regular physical exercise can reduce resting BP and induce post-exercise hypotension, which is a reduction in BP below resting values after exercise training [28]. Therefore, HIIT and MICT have a similar effect of reducing BP. Both HIIT and MICT can be applied as effective exercises for rehabilitation for individuals with SUD in terms of reducing BP.

### Flexibility and vital capacity improved

HIIT, MICT or Tai Chi exercise for different types of participants can improve flexibility [29], which was consistent with the results of this study. The results showed that the two kinds of exercises have similar promoting effects on muscle flexibility.

In this study, VC in the HIIT group increased by 496 ml, whereas that in the MICT group increased by 514 ml. These results demonstrated that improvements in VC were similar. Some preliminary evidence suggests that Tai Chi exercise may increase lung function [30], because Tai Chi combines breathing with movement closely to improve breathing efficiency. Furthermore, another study showed that HIIT and MICT are both effective for improving lung function [31]. HIIT is at least as effective as MCT for improving functional capacity and quality of life measures in patients with pulmonary disease[31]. Therefore, the experimental results were similar to those of some studies, which proved that both HIIT and MICT have similar effects on improving lung VC.

### HIIT is better in strength development

The results of this study showed that the grip strength of the HIIT group increased by 5.48 kg, whilst that of subjects in the MICT group increased by 4.68 kg. The number of push-ups in the HIIT group increased by seven repetitions, whilst that in the MICT group increased by less than five repetitions. These results showed that the improvement in grip strength in the two groups was similar, but the improvement in push-ups in the HIIT group was better than that in the MICT group. Both groups increased (*P* < 0.05) muscle strength, whereas greater muscle strength was gained in the HIIT group. The possible explanation is the training content of the HIIT group includes resistance training [32], which can effectively improve muscle strength. This result has been supported in a previous study. A study reported that HIIT is better at improving upper limb, waist and abdominal strength for individuals with SUD compared with MICT [33].

Interestingly, increased muscle strength was also observed in the MICT group. Some preliminary evidence suggests that Tai Chi exercise may increase the body’s muscle strength [34]. However, another study found that Tai Chi has no advantages in improving muscle strength [27]. Whether Tai Chi intervention improved the strength in the MICT group is unclear, because subjects in the MICT group not only practiced Tai Chi but also engaged in medium-intensity broadcast exercise.

### Does HIIT training have a better effect than MICT?

After 12 months of exercise training, the results showed that HIIT and MICT training had the same effect on the physical fitness of individuals with SUD. The results were not consistent with the previous hypothesis. In general, the greater the intensity of exercise, the more profound the stimulation to the human body, and the more significant the training effect is. However, the differences in physical fitness and craving level between the two groups were not significant. In terms of standing on one leg with eyes closed, the benefits of the subjects in the control group were even better than those in the experimental group, thereby indicating that Tai Chi has a better effect on improving balance ability. The advantage of HIIT over MICT is mainly in terms of cardiovascular function [35], but there is no evidence to support that HIIT training is superior to MICT.

Our results showed the same positive effects on VC, grip strength, sit-and-reach, choice reaction time, one-leg standing with eyes closed and push-up in the two groups. Notably, craving levels decreased in the two groups, which proved the correlation between physical fitness and craving level. Several studies have also demonstrated the same effect of HIIT and MICT. In the exercise intervention with HIIT for type 2 diabetes over 12 weeks, another study found no significant difference in the test indicators of positively altering body fat and increasing peak power output, glucose control, cardiovascular risk and microvascular complication markers in the HIIT and MICT groups [15]. In an exercise intervention for obese men, HIIT was verified as an equally effective exercise mode for improving 24 h glycaemic control in overweight and obese adults compared with MICT [36]. However, Haykowsky et al. [37] showed that HIIT is superior to MICT in terms of improving VO_2_ peak for patients with congestive heart failure, with similar effects on left ventricular function and exercise compliance. Similarly, in patients with coronary heart disease, studies demonstrated that HIIT has greater effect on ventilatory threshold and VO_2_ peak compared with MICT, with similar effects on BP [38].

The advantages and disadvantages of HIIT and MICT remain uncertain, but many studies believe that these two training methods produce equal benefits. There may be several reasons for this phenomenon. Firstly, patients with different symptoms are suitable for varying training methods, and different training methods can produce varying benefits at different stages of the disease. For example, long-term continuous aerobic exercise training plays an important role in maintaining the health and well-being of patients with cardiovascular disease, including the potential to maintain self-care ability and clinical benefits during aging [39]. Short HIIT is useful in the initial and improved stages of cardiac rehabilitation, whilst MICT or HIIT regimens are more appropriate for the improvement and maintenance stages, as they have higher physiological stimulation [21]. Secondly, the correlation between the physical benefits and the amount of exercise generated by HIIT and MICT is greater than the intensity of exercise. Martin J. Gibala et al. [40] compared the six-week HIIT based on Wingate with traditional endurance training; they found a reduction in the weekly training amount (90 % reduction in the HIIT group) and time input (67 % decrease in the HIIT group), but the training had similar improvement on various indexes of skeletal muscle and cardiovascular adaptation. Related studies focus more on the difference between these two training methods and less on the comparison of the total amount of exercise in the same or different situations. Thirdly, no study has explored the optimal intensity of HIIT training. Different intensities of HIIT training may have varying effects on various subjects. Although research suggested that the two training methods have similar effects, as individuals with SUD lose interest to MICT, HIIT and MICT can be alternatively arranged to maintain training interest.

## Conclusions

This study proved that HIIT and MICT intervention are both effective training methods to improve physical fitness in individuals with SUD. The physical fitness of hand grip, sit-and-reach, push-up, balance and reaction time improved while craving level decreased in both groups.

## Limitations

The participants were selected from a Shanghai mandatory detoxification and rehabilitation centre. As physical activity is beneficial for drug dependents physically and mentally, exercise as a supplementary treatment is one of the drug rehabilitation treatments listed in Shanghai detoxification and rehabilitation centres. All substance dependents need to participate in physical activity. Therefore, a participant that does not engage in sports could hardly be found. As a result of this limitation, we failed to confirm the level of benefits brought by exercise training.

In general, the exercise intensity of HIIT is 90–95 % HRmax [41], while the exercise intensity of the experimental group in this study was around 80–85 % HRmax. Most of the subjects belonged to the low-exercise group, so their physical conditions were weak after taking illicit drugs, accounting for the difference in exercise intensity. Considering the exercise risk of the subjects, the optimal exercise intensity could not be achieved. The actual exercise intensity in the HIIT group may not be the optimal intensity to stimulate the physical function of individuals with SUD, which may be one of the factors that caused no significant difference between the HIIT group and MICT group.

The long-term effect of HIIT training for individuals with SUD remains unclear, and the mechanism underlying HIIT training needs further confirmation. In future studies on exercise intervention, the exercise intensity of HIIT will be further enhanced to maintain 90 % HRmax or higher, and the interval time will be strictly controlled.

## Data Availability

The datasets used and/or analyzed during the current study are available from corresponding author on reasonable request.

## Abbreviations

HIIT: High-intensity Interval Training
MICT: Moderate-intensity Continuous Training
SUD: Substance Use Disorder
BP: Blood Pressure
SBP: Systolic Blood Pressure
DBP: Diastolic Blood Pressure
VC: Vital Capacity
VAS: Visual Analog Scale
ATS: Amphetamine-type Stimulant
MA: Methamphetamine
SCRC: Shanghai Compulsory Rehabilitation Center
DSM-IV: Diagnostic and Statistical Manual of Mental Disorders Criteria
CRT: Choice Reaction Time
